# Novel lncRNA lncFAM200B: Molecular Characteristics and Effects of Genetic Variants on Promoter Activity and Cattle Body Measurement Traits

**DOI:** 10.3389/fgene.2019.00968

**Published:** 2019-10-09

**Authors:** Sihuan Zhang, Zihong Kang, Xiaomei Sun, Xiukai Cao, Chuanying Pan, Ruihua Dang, Chuzhao Lei, Hong Chen, Xianyong Lan

**Affiliations:** ^1^College of Animal Science and Technology, Northwest A&F University, Yangling, China; ^2^College of Animal Science and Technology, Yangzhou University, Yangzhou, China

**Keywords:** bovine, lncFAM200B, muscle development, promoter, body measurement traits

## Abstract

Skeletal muscle is one of the three major muscle types in an organism and has key roles in the motor system, metabolism, and homeostasis. RNA-Seq analysis showed that novel lncRNA, *lncFAM200B*, was differentially expressed in embryonic, neonatal, and adult cattle skeletal muscles. The main aim of this study was to investigate the molecular and expression characteristics of *lncFAM200B* along with its crucial genetic variations. Our results showed that bovine *lncFAM200B* was a 472 nucleotide (nt) non-coding RNA containing two exons. The transcription factor binding site prediction analysis found that *lncFAM200B* promoter region was enriched with SP1 transcription factor, which promotes the binding of myogenic regulatory factor MyoD and DNA sequence. The mRNA expression analysis showed that *lncFAM200B* was differentially expressed in embryonic, neonatal, adult bovine muscle tissues, and the *lncFAM200B* expression trend positively correlated with that of *MyoG* and *Myf5* in myoblast proliferation and differential stages. To identify the promoter active region of *lncFAM200B*, we constructed promoter luciferase reporter gene vector pGL3-Basic plasmids containing *lncFAM200B* promoter sequences and transfected them into 293T, C2C12, and 3T3-L1 cells. Our results suggested that *lncFAM200B* promoter active region was from −403 to −139 (264 nt) of its transcription start site, covering 6 SP1 potential binding sites. Furthermore, we found a novel C-T variation, named as SNP2 (ERZ990081 in European Variation Archive) in the promoter active region, which was linked to the nearby SNP1 (rs456951291 in Ensembl database). The genotypes of SNP1 and combined genotypes of SNP1 and SNP2 were significantly associated with Jinnan cattle hip height. The luciferase activity analysis found that the SNP1-SNP2 haplotype CC had the highest luciferase activity, which was consistent with the association analysis result that the combined genotype CC-CC carriers had the highest hip height in Jinnan cattle. In conclusion, our data showed that *lncFAM200B* is a positive regulator of muscle development and that SNP1 and SNP2 could be used as genetic markers for marker-assisted selection (MAS) breeding of beef cattle.

## Introduction

Long non-coding RNA (lncRNA) is an important class of non-coding RNAs (ncRNAs), which are involved in a variety of biological processes. LncRNAs are usually greater than 200 nucleotide (nt) in length, mostly were transcribed by RNA polymerase II, and some were transcribed by RNA polymerase III. Similar to mRNAs, the expression of lncRNAs have obviously temporal (the same tissue on different development stages) as well as the spatial (different tissues) specificity. LncRNA gene has its own promoter, which can be recognized by specific transcription factors. In the last decade, lncRNAs have been showed to have multiple functions in many developmental processes, such as regulating gene expression by transcriptional, post-transcriptional, or epigenetic regulation (Yan et al., 2017; Fernandes et al., 2019). Besides, lncRNAs can serve as the sponges for miRNAs to relieve the repression of miRNAs on their target genes (Sun et al., 2016). Although the biological functions of lncRNAs are very important, their sequence conservation is low among species. Thus, it is important to understand the role of novel lncRNAs in various biological processes in different species.

Skeletal muscles account for about 40% of human body weight, which are not only the dynamic part of the motor system but also play a key role in organism metabolism and homeostasis (Li et al., 2018). Skeletal muscles are composed primarily of multinucleated myotubes, which were originally derived from myogenic progenitor cells (MPCs). MPCs are destined to become myoblasts, which subsequently turn into myotubes after proliferation, differentiation, and fusion (Li et al., 2018). This process is regulated by a variety of transcription factors and epigenetic regulators such as the myogenic regulatory factors myogenic differentiation 1 (MyoD), myogenin (MyoG), myogenic factor 5 (Myf5), and myosin heavy chain 3 (MYH3) (Bharathy et al., 2013). Recently, with the rapid development of sequencing technology, an increasing number of studies found that lncRNA played a crucial role in the development of muscle (Yu et al., 2017; Zhu et al., 2017; Li et al., 2018). In cattle, the lncRNA sequencing showed that lncRNAs were crucial in muscle development (Billerey et al., 2014; Sun et al., 2016; Liu et al., 2017). Although the functions of some lncRNAs such as *lncMD*, *lncYYW*, and *lnc133b* in bovine muscle development have been identified, the roles of numerous lncRNAs are still mysteries to be explored (Sun et al., 2016; Jin et al., 2017; Yue et al., 2017).

Muscle development is one of the main factors that affect cattle growth, and thus, ultimately influences the production economic benefits. Thus, this issue has attracted huge attention in the beef cattle breeding industry. Nowadays, marker-assisted selection (MAS) is a rapid and efficient breeding method, which is based on crucial genetic variation markers (Cui et al., 2018; Chen et al., 2019). Thus, finding muscle development associated genetic variation markers is very important for beef cattle MAS breeding. Given the important role of lncRNA, we think that it would be feasible to screen genetic variations in the muscle development associated lncRNAs region.

Sun et al. (2016) using Ribo-Zero RNA-Seq identified the lncRNA landscape of bovine embryonic, neonatal, and adult skeletal muscles. Within these three developmental stages, 401 differentially expressed lncRNAs were revealed, which included *lncMD* and some new lncRNAs (Sun et al., 2016). In these newly identified lncRNAs, NONBTAT022788 was mapped to the first intron and the second exon (sequence identity is 100%) of *Bos taurus FAM200B* gene (NCBI Reference Sequence: AC_000163.1), thus we aptly renamed it as *lncFAM200B*. In this study, we focused on *lncFAM200B* as it was differentially expressed in bovine embryonic, neonatal, and adult skeletal muscle [the fragments per kilobase of exon per million fragments mapped (FPKM) of *lncFAM200B* were 15.72, 0.41, and 5.73, respectively]. Based on the RNA-Seq results, we speculated that *lncFAM200B* probably plays an important role in the development of bovine skeletal muscle.

Therefore, in this study, we investigated the sequence and expression characteristics of bovine *lncFAM200B* and further, we identified the functional genetic variations in *lncFAM200B* gene. These results would lay the foundation for the function research of *lncFAM200B* and provide scientific data for beef cattle breeding.

## Materials and Methods

All experiments in this study were approved by the Faculty Animal Policy and Welfare Committee of Northwest A&F University (no.NWAFAC1008). The care and use of experimental animals is in full compliance with local animal welfare laws, guidelines, and policies.

### Animal Tissue Samples Collection

To explore the expression profile of *lncFAM200B*, multiple tissue samples from Qinchuan steers at three different developmental stages: embryos of about 3 months old, newborns within 1 week, and adults of about 24 months old were collected from Shaanxi Kingbull Livestock Co., Ltd. (Baoji, China). For sampling at each of the developmental stages, three individuals were used. For each neonatal and adult individual, seven types of tissue samples were collected (heart, liver, spleen, lung, kidney, skeletal muscle, and fat tissue). For embryonic stage, only six kinds of tissue samples were collected (without fat). All samples were frozen immediately in liquid nitrogen and stored at −80°C.

### Total RNA Isolation, cDNA Synthesis, and RACE Experiments

Total RNA was isolated from samples using TRIzol reagent (TaKaRa, Dalian, China). The quality of total RNA was evaluated by 1% agarose gel electrophoresis and NanoDrop 2000 spectrophotometer (Thermo Fisher Scientific, Waltham, MA, USA). Then PrimeScript™ RT reagent Kit with gDNA Eraser (TaKaRa, Dalian, China) was used to synthesize complementary DNA (cDNA), which was used as template for quantitative reverse-transcription PCR (qRT-PCR) or full-length amplification of *lncFAM200B*.

Rapid amplification of cDNA ends (RACE) experiments were carried out to identify the full-length of bovine *lncFAM200B* using bovine fetus skeletal muscle cDNA as template. The 3′RACE was done using PrimeScript™ RT reagent Kit (TaKaRa, Dalian, China) and 3′ RACE universal primers Q_T_, Q_O_, and Q_I_ as described in Scotto-Lavino et al. (2006). The 5′ RACE was done using SMARTer® RACE 5′/3′ Kit (Clontech, Palo Alto, CA, USA) according to the user manual and the previous study (Sun et al., 2016). The 3′ RACE and 5′ RACE specific primers for *lncFAM200B* were designed based on the sequence obtained from RNA-Seq (**Table 1**). Then the full-length of bovine *lncFAM200B* was obtained through sequences assembly based on the results of 3′ and 5′ RACE.

**Table 1:** Primers in this study.

Primers	Primer sequences (5’→3’)	Sizes (bp)	Purpose
qlncFAM200B-F	CCACTTCAAGGAAGTTCCA	93	qRT-PCR
qlncFAM200B-R	TTGTGTTGGTAGCTTGACTA		
GAPDH-F	AAAGTGGACATCGTCGCCAT	116	qRT-PCR
GAPDH-R	CCGTTCTCTGCCTTGACTGT		
MYOG-F	CCAGTACATAGAGCGCCTGC	183	qRT-PCR
MYOG-R	AGATGATCCCCTGGGTTGGG		
MYOD-F	GAACACTACAGCGGCGACTC	126	qRT-PCR
MYOD-R	GCTGTAGTAAGTGCGGTCGT		
MYH3-F	TGCTCATCTCACCAAGTTCC	150	qRT-PCR (Sun et al., 2016)
MYH3-R	CACTCTTCACTCTCATGGACC		
MYF5-F	ACTACTATAGCCTGCCGGGG	238	qRT-PCR
MYF5-R	GGCAATCCAGGTTGCTCTGA		
3’RACE-F	GCTTCCCATCAGAAAGTATCAGGA	141	3*’* RACE
5’RACE-R1	TGCTAAACTGCTGGCTGACACTGGA	295	5*’* RACE
5’RACE-R2	TTCCTTGAAGTGGTGGATTC	268	5*’* RACE
Full length-F	GGTGTTGAGTAGGGAATGG	472	Full-length cloning
Full length-R	TTGTGTTGGTAGCTTGACTACG		
pET-28a-F	CTCCGTCGACAAGCTTGGTGTTGAGTAGGGAATGG	504	Prokaryotic expression
pET-28a-R	GGTGGTGGTGCTCGAGTTGTGTTGGTAGCTTGACTACG		
pGL3-1F	TATCGATAGGTACCGACAACATAGCAGATAATTCGAGTGT	2787	Luciferase reporter system construction for promoter active region identification
pGL3-2F	TATCGATAGGTACCGGCCAACTTTGGAGACCACTT	1994	
pGL3-3F	TATCGATAGGTACCGAATCGGTGGACTGCTAACCT	1143	
pGL3-4F	TATCGATAGGTACCGTCAGCATCACCAGTCACCAAC	744	
pGL3-5F	TATCGATAGGTACCGGCGAGAAAAGGAAACACCGC	480	
pGL3-6F	TATCGATAGGTACCGGGTTAGGCGGGAGGCTTGA	296	
pGL3-R1	GCAGATCTCGAGCCCTCCCCCAGATCTCAAGGGAG		
SNP-F	GTCTCCTCCTGCCTTCAATCT	626	SNP screening
SNP-R	CGAGCGCCAGTGTACCTC		
pGL3-SNP-F	TAGCCCGGGACTCGAGTCTCCTCCTGCCTTCAATCT	594	Construction of luciferase reporter system of SNP1-SNP2 haplotypes
pGL3-SNP-R	CCGGAATGCCAAGCTTCGAGCGCCAGTGTACCTC		
pGL3-SNP1-A-F	TCGCGTGTGGCCGAGAGGGGCGGCCCGGCCA		
pGL3-SNP1-A-R	TGGCCGGGCCGCCCCTCTCGGCCACACGCGA		
pGL3-SNP2-T-F	CTGCTTGATTGGTACTAGCCTCTTCTCCGCT		
pGL3-SNP2-T-R	AGCGGAGAAGAGGCTAGTACCAATCAAGCAG		

### The Sequence Features Analyses and Functional Prediction of Bovine *lncFAM200B*

The coding potential was predicted on Coding Potential Calculator (CPC) website (Kong et al., 2007). The known protein-coding genes CCAAT enhancer binding protein alpha (*C/EBPα*) and lncRNA H19 imprinted maternally expressed transcript (*H19*) were also calculated as control. NCBI-Open Reading Frame Finder (ORF Finder) was used to analyze the open reading frame (ORF) of *lncFAM200B*. The prokaryotic expression system was used to detect the protein coding ability of *lncFAM200B*. The full length of bovine *lncFAM200B* and enhanced green fluorescent protein (*EGFP*) were cloned into vitro prokaryotic expression system pET-28a vector using *Xho*I and *Hin*dIII restriction enzymes and In-Fusion® HD Cloning Kit (TaKaRa, Dalian, China) (Li et al., 2016). The miRDB (http://www.mirdb.org/) was used to predict the interacting miRNAs, and AliBaba2.1 (http://gene-regulation.com/pub/programs/alibaba2/index.html) was used to predict the transcription factors that may bind to the promoter region of *lncFAM200B*.

### Quantitative Reverse-Transcription PCR

The qRT-PCR was performed to detect the expression of *lncFAM200B* in tissues. The housekeeping gene glyceraldehyde-3-phosphate dehydrogenase (*GAPDH*) was used as internal control. The primers for qRT-PCR were listed in **Table 1**. The qRT-PCR was performed in a Bio-Rad CFX Manager 3.1 (Bio-Rad Laboratories, Hercules, CA, USA) using SYBR® Premix Ex Taq™ II (Tli RNaseH Plus) (TaKaRa, Dalian, China) (Kang et al., 2019a). All samples were detected in triplicate. The relative expression levels of mRNA in tissue samples were calculated using the 2^−∆∆Ct^ method (Livak and Schmittgen, 2001). The correlations between genes were calculated using Pearson correlation analysis, and the differences between samples were calculated using Student *t*-test (Chen et al., 2018).

### Cell Culture, Plasmids Construction, and Transfection

The procedure for separating bovine myoblast from skeletal muscle was the same as the previous study of our lab (Sun et al., 2016). Then cells were cultured in incubator at 37°C with 5% CO_2_. The proliferation medium for myoblast contains 80% Dulbecco’s Modified Eagle Medium (DMEM), 20% fetal bovine serum (FBS), penicillin (10 U/ml), and streptomycin (10 mg/ml). When myoblast start to fuse, the proliferation medium was replaced by differential medium, which contains 2% horse serum, penicillin (10 U/ml), streptomycin (10 mg/ml), and DMEM. The RNA of the myoblast was collected using TRIzol reagent (TaKaRa, Dalian, China) at proliferation and differential stages. Mouse C2C12 myoblast cells, mouse 3T3-L1 embryo fibroblast, and human embryonic kidney 293T cells were used to uncover the active region of *lncFAM200B* promoter or single nucleotide polymorphisms (SNPs) effects on promoter activity. They were grown in 10% FBS, 90% DMEM, penicillin (10 U/ml), and streptomycin (10 mg/ml) medium.

To investigate the active region of *lncFAM200B* gene promoter, six fragments of the *lncFAM200B* promoter region were amplified and cloned into the pGL3-Basic vector (Promega, Madison, WI, USA) using *Sac*I and *Sma*I restriction enzymes (**Table 1**). These constructed plasmids were named as pGL3-pro1 (2,787 base pairs [bp]), pGL3-pro2 (1,994 bp), pGL3-pro3 (1,143 bp), pGL3-pro4 (744 bp), pGL3-pro5 (480 bp), and pGL3-pro6 (296 bp) according to their sequence length. The largest fragment (2,787 bp) spans from −2,446 nt to +310 nt of the *lncFAM200B* transcription start site. Additionally, four plasmids termed as pGL3-CC (SNP1-C and SNP2-C), pGL3-CT (SNP1-C and SNP2-T), pGL3-AC (SNP1-A and SNP2-C), and pGL3-AT (SNP1-A and SNP2-T) were constructed using overlap PCR to detect the effects of haplotype on promoter activity (**Table 1**). The vector pRL-TK was used as internal reference in the luciferase reporter system. The pGL3-Control and empty pGL3-Basic were used as positive and negative control, respectively (Xu et al., 2018; Kang et al., 2019b).

The plasmids were transfected into cells using Lipofectamine 2000 (Invitrogen, Carlsbad, CA, USA). Before transfection, cells were seeded into 96-well plate. When cells covered 80% of the culture plate bottom, the plasmids were transient transfected according to the manufacturer’s protocol. To normalize the transfection efficiency, the pRL-TK was transfected with constructed plasmids, and the transfection ratio of constructed plasmids and pRL-TK was 50:1 (Kang et al., 2019b). All transfections were carried out in triplicate. After 36 h, the cells were lysed, and the luciferase activity was measured using BHP9504 microporous-plate luminescence analyzer (Hamamatsu Photons Technology, Beijing, China). The relative luciferase activity of different promoter fragments were normalized by renilla luciferase activity (Xu et al., 2018; Kang et al., 2019b). The relative luciferase activity was represented by mean ± standard deviation. The one-way ANOVA and Bonferroni multiple comparisons were used to analyze the difference between groups (Yang et al., 2019).

### Genetic Variation Analyses of Bovine *lncFAM200B* Promoter Region

A total of 352 female cattle from four breeds were used in this study to identify the novel genetic variations in bovine *lncFAM200B* promoter region. The samples of Qinchuan cattle (*n* = 139), Jinnan cattle (*n* = 121), Nanyang cattle (*n* = 67), and Ji’an cattle (*n* = 25) were randomly collected from Shaanxi, Shanxi, He’nan, and Jiangxi provinces, respectively. The detailed information and records of body measurement traits for the cattle were the same as the published papers (Zhang et al., 2015; Jin et al., 2018). The blood DNA samples were isolated using high salt-extraction method (Aljanabi and Martinez, 1997). The primers (SNP-F and SNP-R) used to identify the genetic variations were designed based on the DNA sequence of bovine *lncFAM200B* gene. All the variations were identified by agarose gel electrophoresis and DNA sequencing (Sangon Biotech, Shanghai, China). After genotyping, the genotypic and allelic frequencies, population genetic diversity indexes [Hardy-Weinberg equilibrium (HWE), heterozygosity (He), effective population size (Ne), polymorphism information content (PIC)] were calculated according to the methods described as Nei (1973) using MSR website (http://www.msrcall.com/) (Wang et al., 2017; Yang et al., 2017). Then the association analyses between genotypes and records of body measurement traits were performed based on the reduced linear model below: Y*_i_* = u + G*_i_* + e, where Y*_i_* was the trait measured data for each animal; u was the over mean for each trait; G*_i_* was the effect of genotype; and e was the random error. Different breeds were analyzed separately. Due to all the cattle were 2−3 years old female and the individuals of the same breed were bred in the same farm, so this model excluded the farm, breed, years old, and sex factors. The linkage disequilibrium (LD) and haplotypes analyses were performed using SHEsis online platform (http://analysis.biox.cn^1^; Cui et al., 2018). The association analyses between genotypes or haplotypes and body measurement traits were performed by one-way ANOVA followed by Bonferroni multiple comparison (three groups) or independent-sample *t*-test (two groups) (Wang et al., 2019).

## Results

### Characterization of Bovine *lncFAM200B*

**Figure 1:**
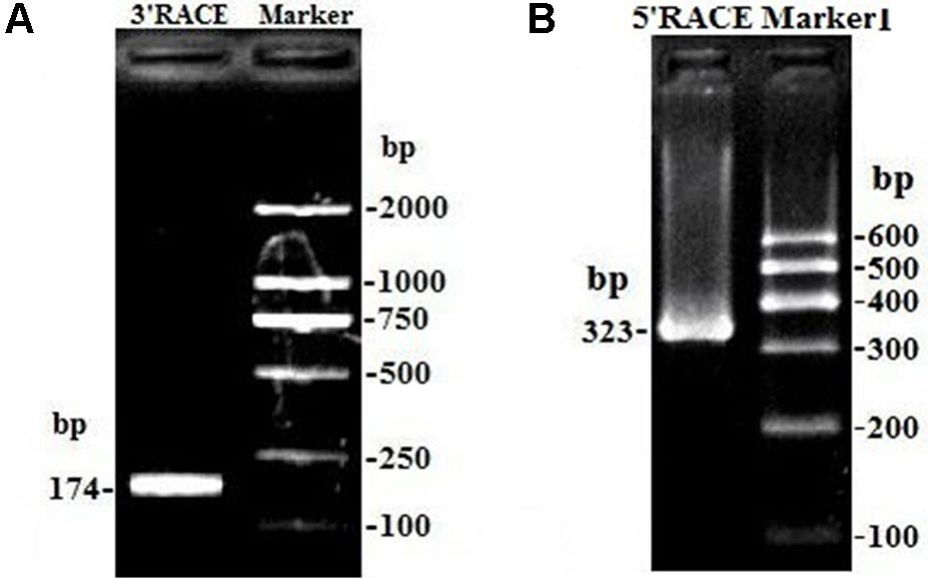
The amplification products of *lncFAM200B* 3*’* RACE and 5*’* RACE. **(A)** the amplification product of *lncFAM200B* 3*’* RACE, 174 bp = 141 bp (*lncFAM200B* sequence) + 15 bp (poly A) + 18 bp (Q_I_). **(B)** the amplification product of *lncFAM200B* 5*’* RACE, 323 bp = 268 bp (*lncFAM200B* sequence) + 33 bp (5*’* RACE adapter) + 22 bp (primer).

**Figure 2:**
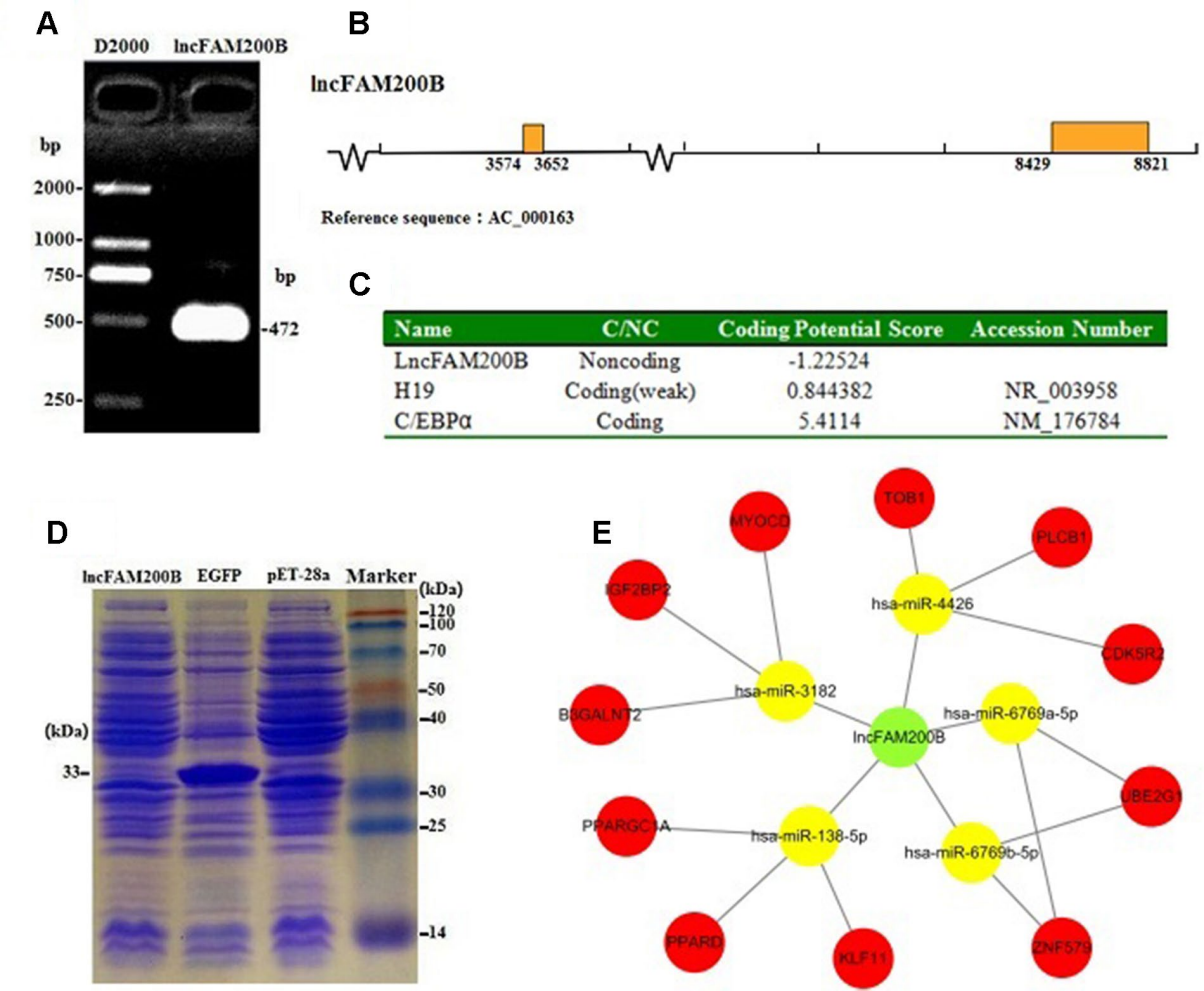
Characterization of bovine *lncFAM200B*. **(A)** The full-length of *lncFAM200B*; **(B)** The distribution mode chart of *lncFAM200B* exons. The box and the line represented the exon and intron, respectively; **(C)** The coding ability prediction of *lncFAM200B* using CPC website; **(D)** The *in vitro* translation system of protein product from *lncFAM200B*; **(E)** The potential interacting miRNAs of *lncFAM200B* and miRNAs target genes.

Due to only partial sequence (369 nt) was obtained by RNA-Seq (Sun et al., 2016), the 5′ and 3′ RACE were carried out to obtain the full length of *lncFAM200B*. The 3′ and 5′ RACE obtained 174 bp and 323 bp sequences, respectively (**Figure 1**). The full-length of bovine *lncFAM200B* was 472 nt and had two exons (**Figures 2A, B**). The protein-coding potential prediction score of bovine *lncFAM200B* in CPC was −1.22524, which was far less than the scores of the known protein-coding genes *C/EBPα* and lncRNA *H19* (**Figure 2C**). Meantime, all the ORFs in *lncFAM200B* were smaller than 100 amino acids, illustrated that the coding ability of *lncFAM200B* was very low (Sun et al., 2016). To ensure the coding ability of *lncFAM200B*, the prokaryotic expression system was implemented and it showed that no protein was being encoded by *lncFAM200B* (**Figure 2D**).

The miRNA prediction analysis uncovered that 8 miRNAs might interact with *lncFAM200B*. Among these miRNAs, 5 miRNA scores were above 60, so we further predicted the target genes of these 5 miRNAs. As a result, some cell proliferation associated genes were uncovered, such as insulin like growth factor 2 mRNA binding protein 2 (*IGF2BP2*) (**Figure 2E**). Furthermore, as it is known that few lncRNAs could interact with their nearby genes, we searched the adjacent genes of *lncFAM200B*. Interestingly, we found that fibroblast growth factor binding protein 1 (*FGFBP1*) was close to *lncFAM200B.* Thus, *lncFAM200B* might interact with *FGFBP1* and affect cell proliferation and differentiation (Xie et al., 2006). The transcription factors binding sites prediction analysis found that within the 3000 bp sequence region upstream of *lncFAM200B*, there were 30 C/EBPα, 7 CCAAT/enhancer binding protein beta (C/EBPβ), and 43 SP1 transcription factor binding sites. Hayashi et al. (2016) found that the area enriched with SP1 was highly prone to promote the binding of MyoD and DNA sequence. Since the MyoD was a crucial transcription factor during muscle cell differentiation, we think that the identified region must be important for the transcription of bovine *lncFAM200B*.

### Expression Profiles of *lncFAM200B* in Bovine Tissues and Myoblasts

**Figure 3:**
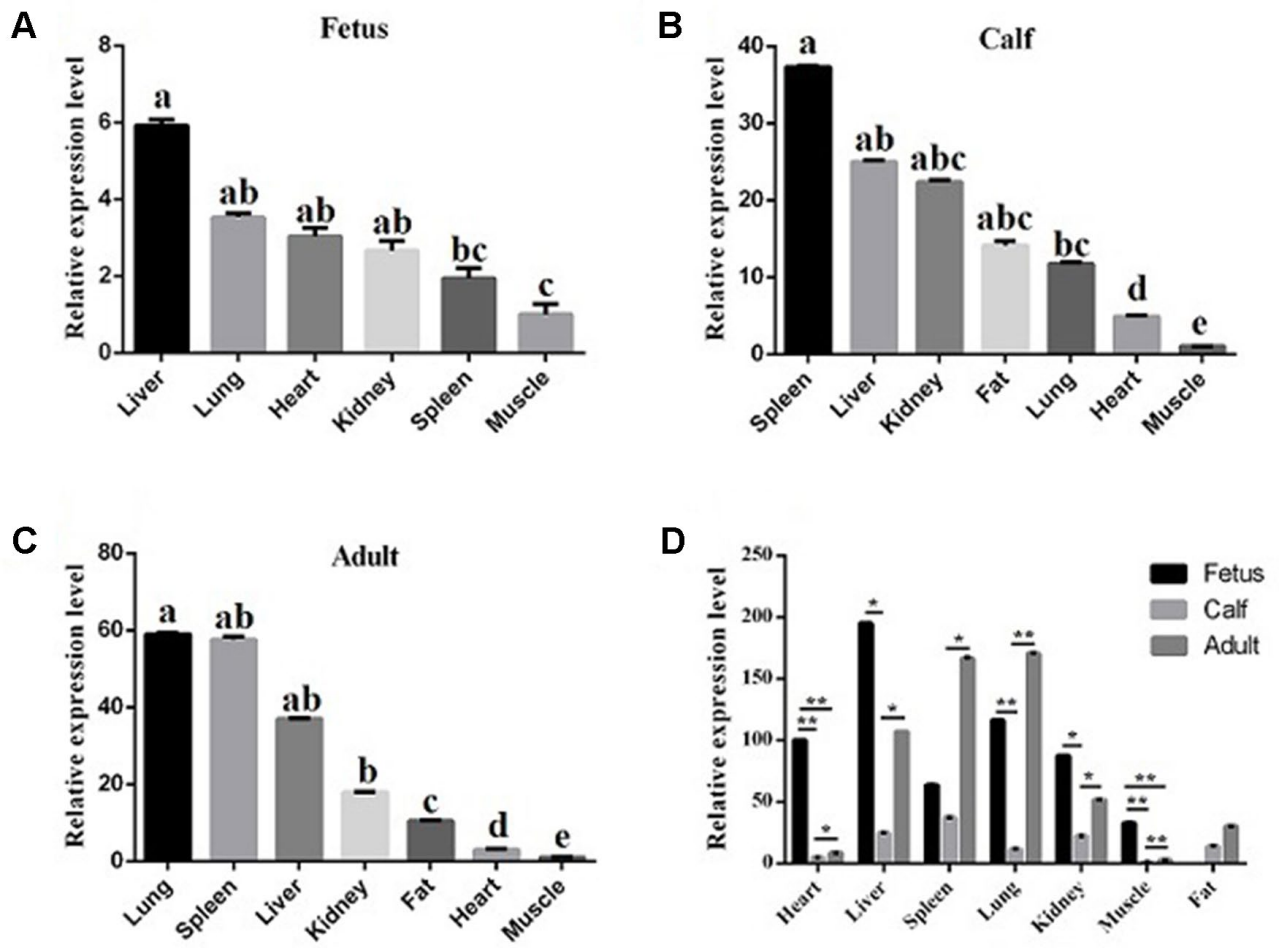
The relative expression levels of lncFAM200B in tissues of Qinchuan cattle. Expression level of lncFAM200B in fetus **(A)**, calf **(B)**, adult, **(C)** tissues **(D)**. **(A**, **B**, **C)** The columns with different superscripts (a, b, c, d, e) within each figure differ significantly at *P* < 0.05 level. **(D)** *P < 0.05; **P < 0.01.

**Figure 4:**
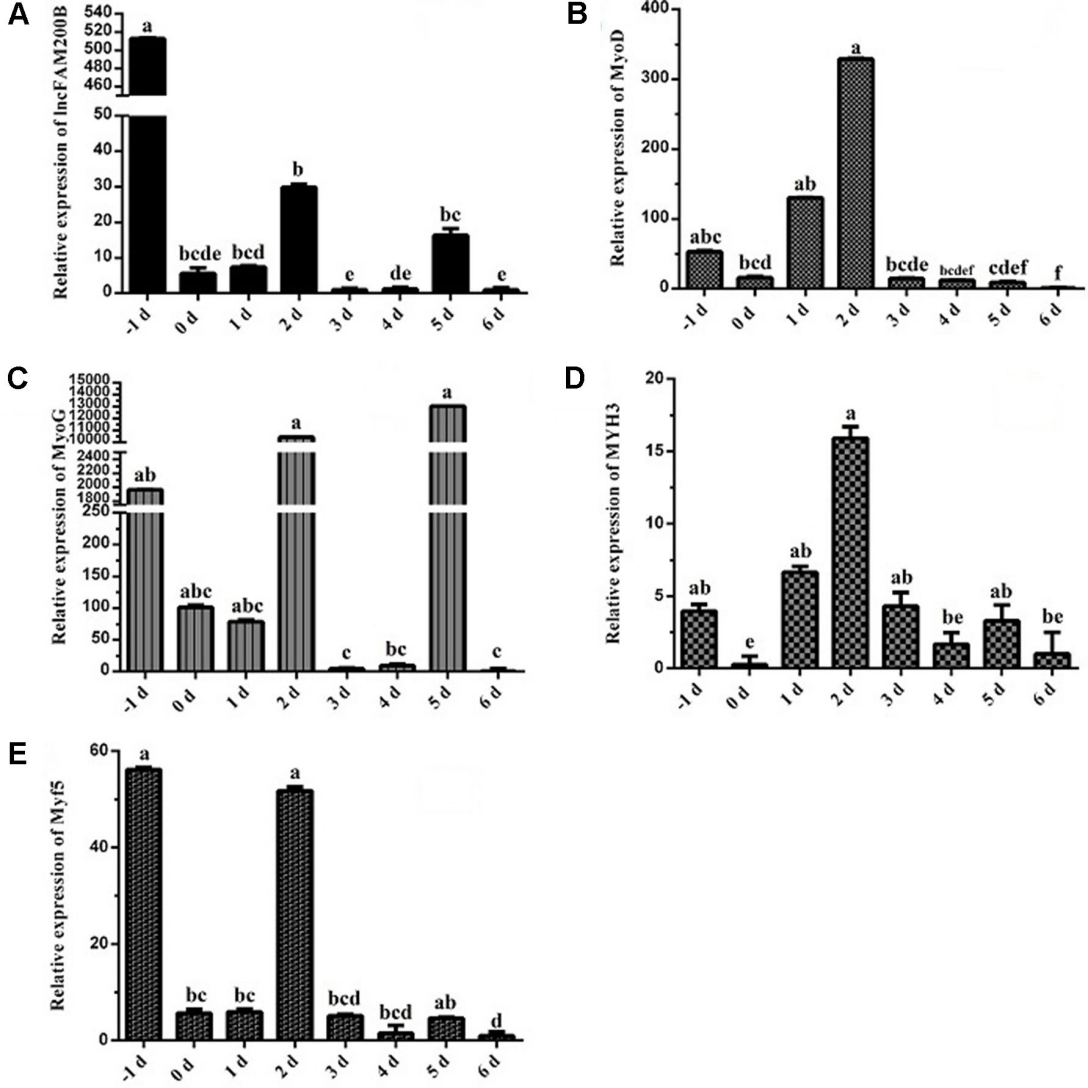
Expression characteristics of *lncFAM200B* and myoblast development associated genes in bovine myoblast. Expression trend of *lncFAM200B* **(A)**, *MyoD* **(B)**, *MyoG* **(C)**, *MYH3* **(D)**, and *Myf5* **(E)** in bovine myoblast cultured in proliferation medium (−1 day) and differentiation medium (0, 1, 2, 3, 4, 5, and 6 days).

To reveal the function of *lncFAM200B*, we investigated the expression profiles in bovine embryonic, neonatal, and adult tissues. In various bovine tissues, *lncFAM200B* was widely expressed in three developmental stages (**Figures 3A–C**). In skeletal muscle, the expression level of *lncFAM200B* was low at each state, but was significantly different among the three developmental stages (**Figure 3D**), which was consistent with the RNA-Seq data. At the cellular level, we detected the expression level of *lncFAM200B*, *MyoD*, *MyoG*, *Myf5*, and *MYH3* genes in myoblast proliferation and differential stages, which were important in the regulation of myoblast development (**Figure 4**). The expression characteristic of *lncFAM200B* showed a significant positive correlation with the expression of *MyoG* (Pearson correlation coefficient = 0.922, *P* = 0.003) and *Myf5* (Pearson correlation coefficient = 0.741, *P* = 0.035) (**Table 2**). These results suggested that *lncFAM200B* might be involved in the development of bovine myoblasts.

**Table 2:** Pearson correlation analyses between the expression of *lncFAM200B* and myoblast development associated genes in proliferation and differentiation states muscle cell.

Gene	*MyoD*	*MyoG*	*MYH3*	*Myf5*
Pearson correlation coefficient	0.527	0.922**	0.442	0.741*
Sig.(2-tailed)	0.179	0.003	0.273	0.035

*P < 0.05; **P < 0.01.

### Identification of Bovine *lncFAM200B* Promoter Active Region

**Figure 5:**
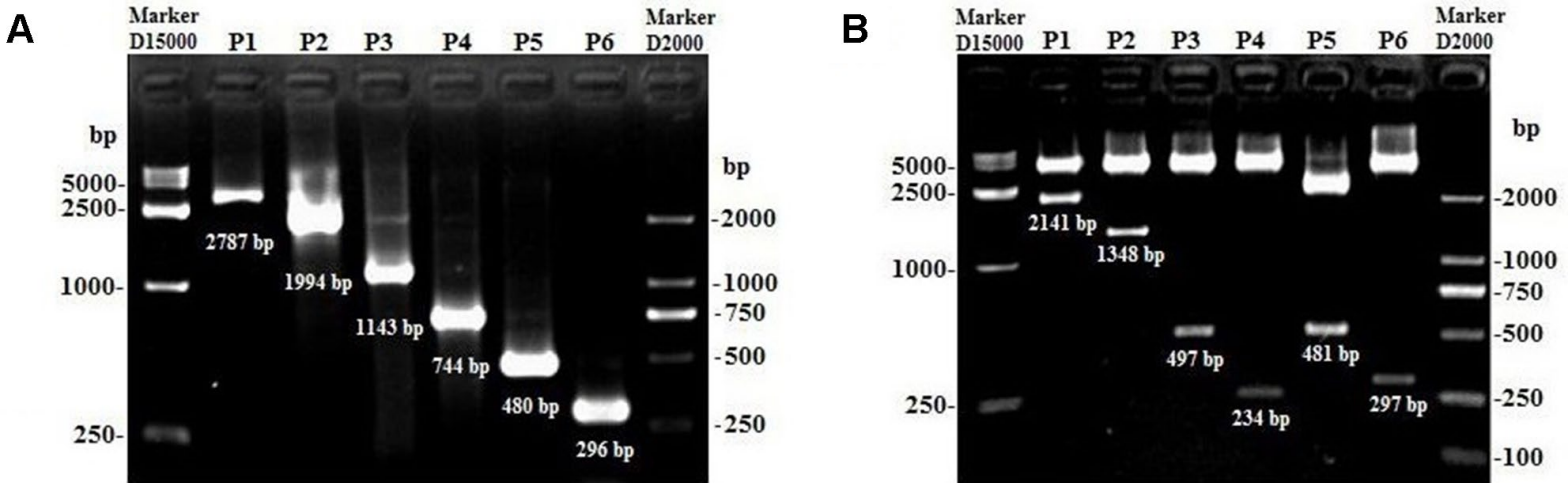
The products of *lncFAM200B* promoter fragments and the identification of the plasmids using different restriction enzymes. **(A)** P1 to P6 represented the PCR products of pGL3-pro1 to pGL3-pro6. **(B)** P1 to P6 represented the recombined plasmids of pGL3-pro1 to pGL3-pro6 digested by different restriction enzymes.

**Figure 6:**
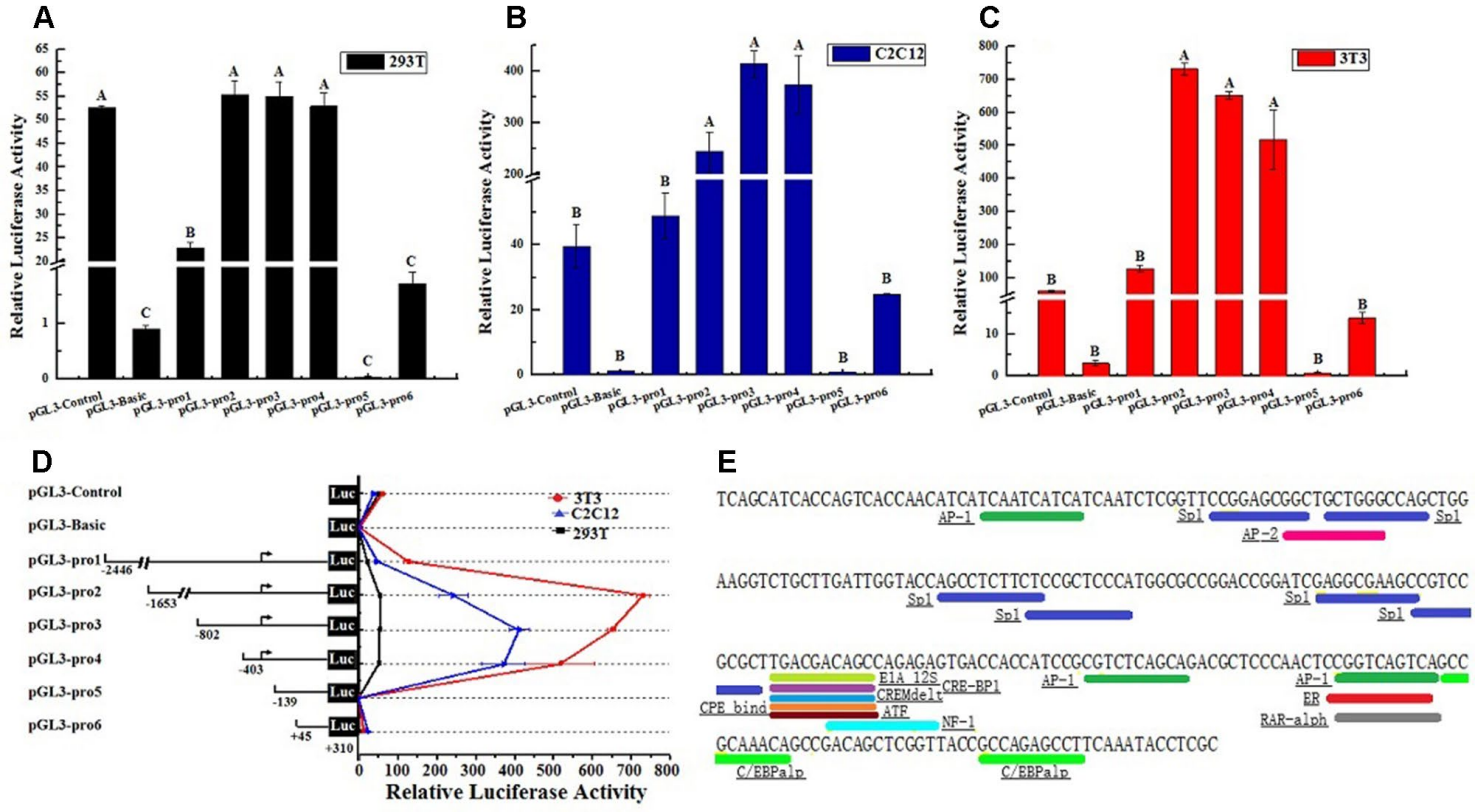
The bovine *lncFAM200B* promoter active region. Relative luciferase activity of different promoter fragments in **(A)** 293T, **(B)** C2C12, **(C)** 3T3-L1 cell lines; **(D)** Relative luciferase activity changed trend; **(E)** Potential transcription factor binding sites in promoter activity region.

Considering the characteristic of *lncFAM200B* promoter region, this study further confirmed the promoter active region of bovine *lncFAM200B*. Six truncated fragments of the promoter region were constructed into pGL3-Basic plasmid and transfected into 293T, C2C12, and 3T3-L1 cells. By restriction enzyme identification and plasmids sequencing analyses, we confirmed that the recombinant plasmids were successfully constructed (**Figure 5**). The detection of double luciferase activity showed that the luciferase activity of different truncated fragments showed the same trend in these three different cell lines (**Figure 6D**). In each cell line, the luciferase activity of positive control (pGL3-Control) was high, but the negative control (empty pGL3-Basic) was low (**Figures 6A–D**), providing the basis for our observations and correct experimental design. The pGL3-pro2, pGL3-pro3, and pGL3-pro4 yielded a significantly stronger luciferase activity compared to the other vectors (*P* < 0.01; **Figures 6A–C**), which suggested that these fragments contained promoter active region. The luciferase activity of the longest fragment pGL3-pro1 was lower than that of pGL3-pro2, pGL3-pro3, and pGL3-pro4 (**Figures 6A–C**), suggesting that there might be inhibitor binding sites in the region (−2,446 to −1,653) of the *lncFAM200B*. Particularly, from pGL3-pro4 to pGL3-pro5, the luciferase activity dramatically decreased (*P* < 0.01; **Figures 6A–C**), which meant that the active region was truncated in pGL3-pro5 and the active region was from −403 to −139 (264 nt) of the *lncFAM200B* transcription start site (**Figure 6D**). Besides, upon the transcription factor binding site prediction, we found 6 SP1 and 2 C/EBPα potential binding sites in the active region (−403 to −139) (**Figure 6E**). Above results suggested that the 264 nt active region was crucial for the expression of bovine *lncFAM200B*.

### Novel Genetic Variations in Bovine *lncFAM200B* Promoter Region

**Figure 7:**
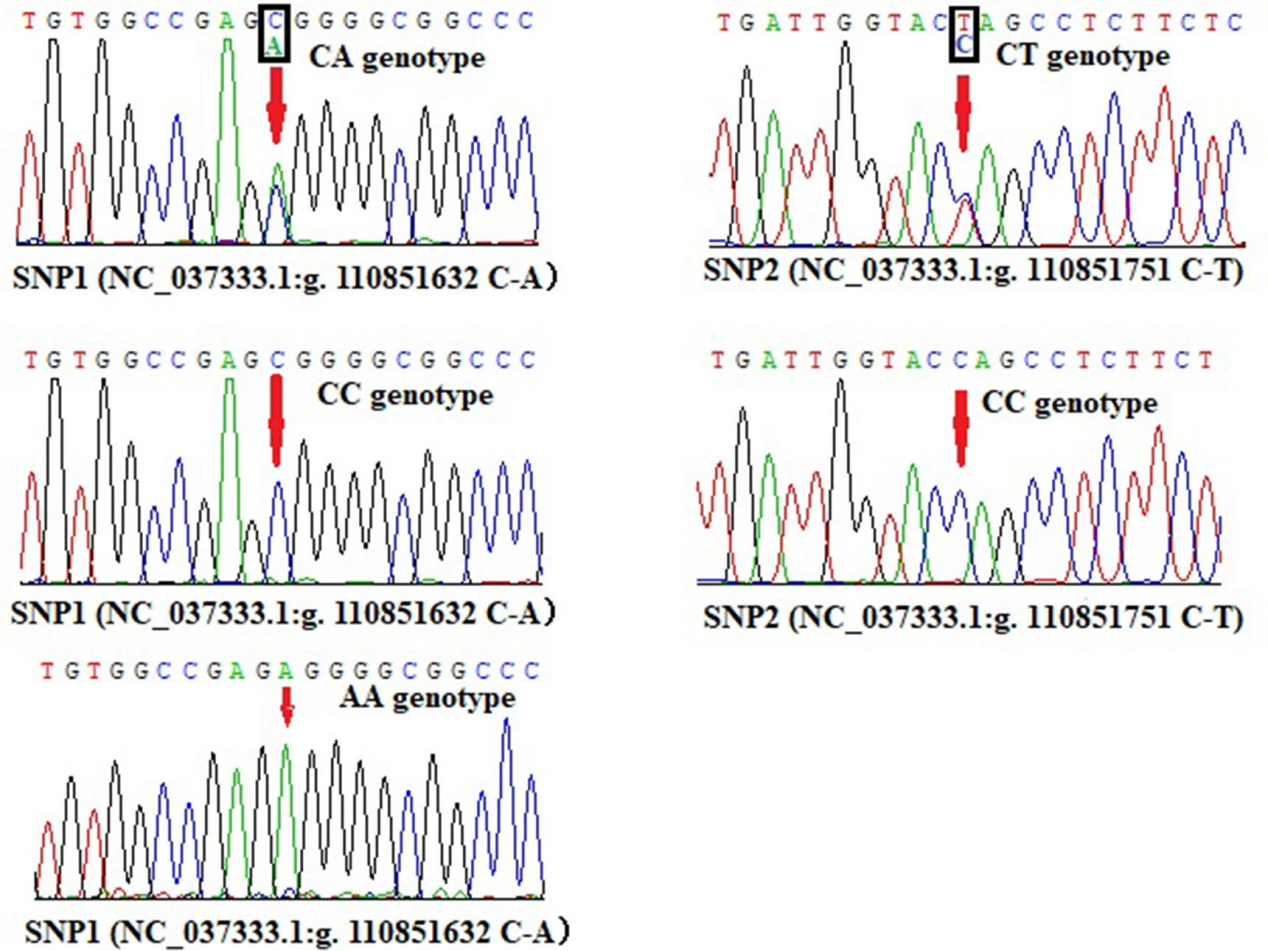
Sequencing maps of SNP1 and SNP2 different genotypes in *lncFAM200B* gene promoter region.

Promoter active region is very important for gene expression, hence we wanted to know whether there are crucial genetic variations in this region. Based on the DNA sequencing results, two SNPs were revealed in the promoter region of bovine *lncFAM200B*, SNP1 (NC_037333.1:g.110851632 C-A, rs456951291 in Ensembl database) and a novel genetic variant SNP2 (NC_037333.1:g.110851751 C-T, ERZ990081 in European Variation Archive) (**Figure 7**). Interestingly, SNP2 was in the promoter active region of bovine *lncFAM200B*.

At SNP1 locus, CC and CA genotypes were identified in cattle (three genotypes were identified in Jinnan cattle). At SNP2 locus, only CC and CT were identified in the four detected cattle breeds (**Table 3**; **Figure 7**). At these two loci, C was the main allele in all the detected cattle breeds. The Chi-squared test showed that these loci were at Hardy-Weinberg equilibrium (*P* > 0.05) in the four populations (**Table 3**). Further, population genetic parameters indicated that the loci were polymorphic but belonged to low (PIC < 0.25) or moderate (0.25 < PIC < 0.50) polymorphisms categories (**Table 3**). Then LD analyses between SNP1 and SNP2 were analyzed in Qinchuan, Jinnan, and Ji’an populations [‘in Nanyang cattle the individual numbers of CA (SNP1 locus) and CT (SNP2 locus) were found to be smaller than 3, so we did not perform the LD analysis and the follow association analysis]. The *D*’ and *r* ^2^ values in Qinchuan (*D*’ = 1.000, *r* ^2^ = 0.735), Jinnan (*D*’ = 0.611, *r* ^2^ = 0.049), and Ji’an (*D*’ = 0.857, *r* ^2^ = 0.532) cattle populations showed these two loci were linked in cattle. The *r* ^2^ reflects the extent of the linkage disequilibrium and *r* ^2^ > 0.33 indicated that there was a sufficiently strong linkage between the two loci. When different genotypes are evenly distributed in the population, the *D*’ > 0.33 can also be used to judge that there was a linkage disequilibrium (Zhao et al., 2007).

**Table 3:** Calculation of the parameters of the genetic variations in bovine *lncFAM200B* promoter region.

Loci/Breeds	Genotype numbers (frequencies)			Allele frequencies		HWE	Population parameters		
SNP1	CC	CA	AA	C	A	*P* values	He	Ne	PIC
Nanyang	65 (0.97)	2 (0.03)	/	0.99	0.01	0.901	0.029	1.030	0.029
Qinchuan	119 (0.86)	20 (0.14)	/	0.93	0.07	0.361	0.134	1.154	0.125
Jinnan	46 (0.38)	58 (0.48)	17 (0.14)	0.62	0.38	0.851	0.459	1.848	0.354
Ji’an	14 (0.56)	11 (0.44)	/	0.72	0.28	0.158	0.343	1.523	0.284
SNP2	CC	CT	TT	C	T	*P* values	He	Ne	PIC
Nanyang	65 (0.97)	2 (0.03)	/	0.99	0.01	0.901	0.029	1.030	0.029
Qinchuan	124 (0.89)	15 (0.11)	/	0.95	0.05	0.501	0.102	1.114	0.097
Jinnan	103 (0.85)	18 (0.15)	/	0.93	0.07	0.377	0.138	1.160	0.128
Ji’an	11 (0.44)	14 (0.56)	/	0.72	0.28	0.052	0.403	1.680	0.322

HWE, Hardy-Weinberg equilibrium; He, heterozygosity; Ne, effective population size; PIC, polymorphism information content.

**Figure 8:**
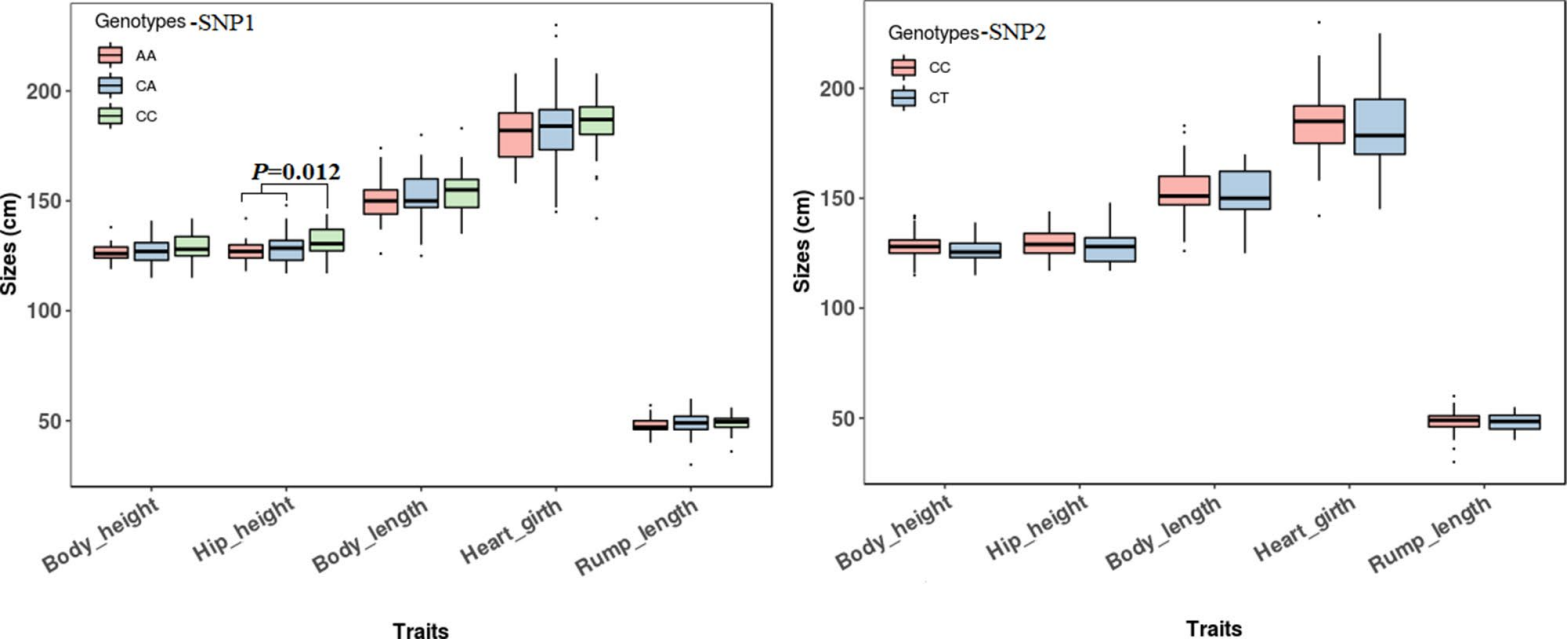
Association of *lncFAM200B* SNPs (left−SNP1; right−SNP2) and body measurement traits of Jinnan cattle.

**Figure 9:**
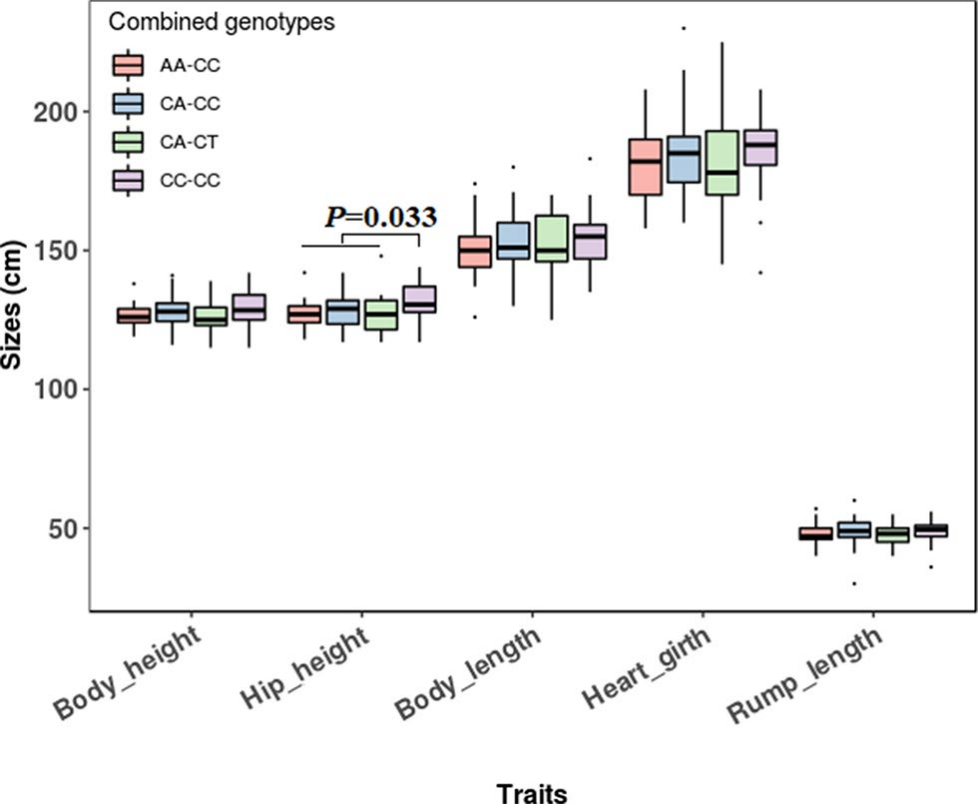
Association of *lncFAM200B* SNP1-SNP2 combined genotypes and body measurement traits of Jinnan cattle.

The association analyses found that the genotypes of SNP1 were significantly associated with the hip height in Jinnan cattle (*P =* 0.012). The hip height of the CC genotype carriers was 131.7 ± 6.7 cm, which was evidently higher than that of CA (128.6 ± 6.5 cm) and AA (127.1 ± 5.4 cm) genotype carriers, but we did not observe any significant difference between CA and AA genotype carriers (**Figure 8**). Besides, at SNP1 and SNP2 loci, the body measurement traits (hip height, body height, body length, heart girth, rump length) of CC genotype carriers were all better than the carriers with the other genotypes in Jinnan cattle (**Figure 8**). Furthermore, the combined genotypes of SNP1 and SNP2 were found to be significantly associated with hip height in Jinnan cattle (*P* = 0.033). The hip height of the CC-CC carriers (132.0 ± 6.6 cm, *n* = 44) was markedly higher than that of CA-CT (127.7 ± 7.9 cm, *n* = 15), CA-CC (128.9 ± 6.0 cm, *n* = 43), and AA-CC (127.1 ± 5.4 cm, *n* = 17) genotype carriers (**Figure 9**). Because we only found one individual with CC-CT and one individual with AA-CT genotype, they were excluded in association analyses (**Table 4**). In Qinchuan and Ji’an cattle, no significant association was found between SNP1, SNP2, or the combined genotypes and the body measurement traits.

**Table 4:** Genotypic frequencies of *lncFAM200B* SNP1-SNP2 combined genotypes in cattle.

Breeds	Sample size(*N*)	SNP1-SNP2 combined genotypes numbers (frequencies)					
		CC-CC	CA-CT	CA-CC	AA-CC	CC-CT	AA-CT
QC	139	119 (0.86)	15 (0.11)	5 (0.03)	/	/	/
Jinnan	121	44 (0.36)	15 (0.12)	43 (0.36)	17 (0.14)	1 (0.01)	1 (0.01)
Ji’an	25	10 (0.40)	10 (0.40)	1 (0.04)	/	4 (0.16)	/

### Influence of the Haplotypes on the Transcriptional Activity of Bovine *lncFAM200B*

**Figure 10:**
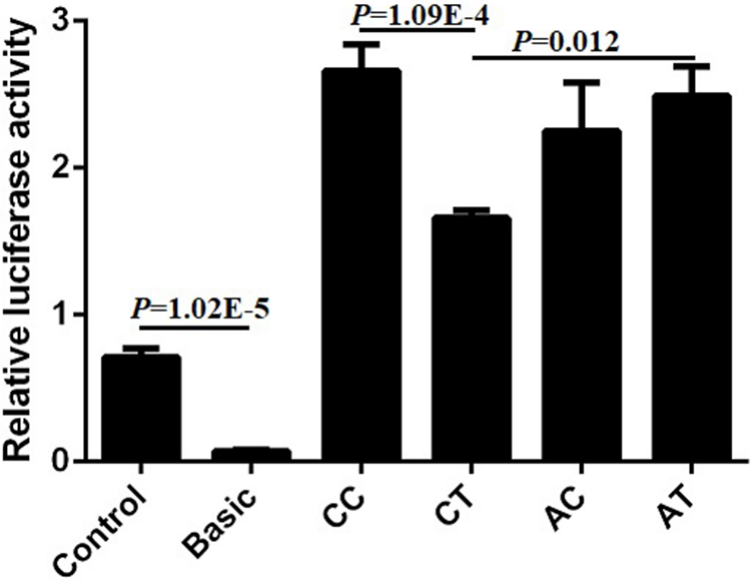
Relative luciferase activity of different haplotypes of bovine *lncFAM200B* SNP1-SNP2 in 293T cell.

Bearing in mind the significant relationship between SNP1 and the combined genotypes with the cattle body measurement traits, we wanted to further investigate the mechanism that contributed to the phenotype. Four plasmids (pGL3-CC, pGL3-CT, pGL3-AC, pGL3-AT) of SNP1 and SNP2 haplotypes were constructed and transfected into commonly used 293T cells to detect the luciferase activity. The luciferase activity of positive control (Control) was significantly higher compared to that of the negative control (empty Basic) and we found that the relative luciferase activity of pGL3-CC was the highest among the four haplotypes in 293T cells. The luciferase activities of pGL3-CC and pGL3-AT were significantly higher than that of the pGL3-CT haplotypes (*P* < 0.05). But no difference was found among the other haplotypes (**Figure 10**). These results suggested that the genotypes of SNP1-SNP2 haplotypes influenced the body measurement traits by regulating the expression of *lncFAM200B*.

## Discussion

With the rapid development of high-throughput sequencing technology, an increasing number of lncRNAs have been discovered in many animal species. Structurally, the lncRNA resembled protein-coding gene with its own promoter, exons, and introns. The *lncFAM200B* was screened from the sequencing results obtained in an earlier study done by Sun et al. (2016). In their study, they implemented strict parameters to identify the lncRNA from the sequencing results such as the number of exons must be ≥2, the size must ≥200 nt, the read number should be >3, the ORF should be no longer than 100 amino acids, and the predicted protein-coding potential should be weak (Li et al., 2016; Sun et al., 2016). Based on their research, we used different methods (RACE, *in vitro* prokaryotic expression system, and protein-coding ability prediction analysis) to further prove that *lncFAM200B* was a novel lncRNA.

Expression analysis found that the expression of *lncFAM200B* positively correlated with the expression of *MyoG* (*P* = 0.003) and *Myf5* (*P* = 0.035). MyoG, a muscle-specific transcription factor, positively regulated the skeletal muscle fiber development, myoblast differentiation, and fusion, and was found to be indispensable for myogenic differentiation (Zammit, 2017). Myf5 is a master regulator belonging to the MRFs family and is known to play a key role in muscle differentiation or myogenesis. *Myf5* is a master gene for the determination of skeletal muscle, which pushes the myogenic precursors into myoblasts (Dimicoli-Salazar et al., 2011). The genes have the same expression pattern may have the same function, such as *MEGF10*, a myogenic regulator of satellite cells in skeletal muscle, shares a similar expression pattern with *MyoG* in muscle regeneration (Park et al., 2014). Thus, we hypothesize that *lncFAM200B* might play a positive role in muscle development.

The molecular markers based on nucleotide sequence variations among individuals, which are the directly reflection of genetic polymorphism in DNA level. Compared to the morphological markers, DNA molecular markers have many advantages. Genomic variations are extremely abundant and are the impetus of biological evolution providing rich material for animal breeding. At different stages of biological development, such as the early disease diagnosis and early animal selection for breeding, the DNA markers can be used. The detection method of DNA genetic variations is simple and rapid. Nowadays, DNA markers are widely used in biological evolution analysis, genetics analysis, diagnosis of genetic diseases and so on (Alidoust et al., 2018). In animal breeding, it is important to explore crucial markers. In cattle, numerous variations have been identified within the protein-coding genes, but only a few studies have uncovered the variations in the non-coding RNA genes (Jin et al., 2018; Yu et al., 2018). In this study, first, we analyzed the SNPs in the promoter region of *lncFAM200B* gene and found that the SNP1 was linked with the promoter active region mutation, SNP2. Importantly, the genotypes of SNP1 and combined genotypes of SNP1 and SNP2 were associated with the hip height in Jinnan cattle.

We attempted to uncover the cause of the above SNP effect on the cattle growth trait. Promoter regulates the activity of gene by affecting the binding of transcription factors and DNA promoter region sequences. Mutations in the gene promoter region will result in gene expression disorder, further resulting in phenotypic changes and disease (Lu et al., 2019). In this study, we used the dual-luciferase reporter system to detect the effects of SNP1 and SNP2 variations on gene expression. In the commonly used 293T cells, haplotype CC showed the highest fluorescence value followed by haplotype AT and both were significantly higher than haplotype CT. The haplotype CC had the highest hip height, which agreed with the luciferase activity data. These results further provided evidence proving that *lncFAM200B* is a positive regulator of muscle development.

## Conclusion

The lncRNA *lncFAM200B* differentially expressed in embryonic, neonatal, and adult bovine skeletal muscles. In myoblast proliferation and differential stages, the expression characteristic of *lncFAM200B* was positively correlated with the expression of *MyoG* and *Myf5*. In *lncFAM200B* active region (−403 to −139 of *lncFAM200B* transcription start site), one novel SNP (SNP2, NC_037333.1:g.110851751 C-T, ERZ990081) was discovered which linked with the nearby SNP1 (rs456951291). The genotypes of the SNP1 and the combined genotypes of SNP1 and SNP2 were significantly associated with the hip height in Jinnan cattle. Interestingly, haplotype CC had the highest luciferase activity and the highest hip height. Our results established that *lncFAM200B* is a positive regulator of muscle development and we believe that our studies will help in advancing the beef cattle MAS breeding program.

## Data Availability Statement

The detailed information of SNP2 can be found in the European Variation Archive database after 2019/12/31. Project: PRJEB33081; Analyses: ERZ990081.

## Ethics Statement

The animal study was reviewed and approved by Faculty Animal Policy and Welfare Committee of Northwest A&F University (no. NWAFAC1008).

## Author Contributions

SZ and XL conceived and designed the experiments. SZ, ZK, and CP performed the experiments. XS provided the RNA-Seq data. XC, RD, CL, HC, and XL collected the DNA samples. SZ and XL analyzed the data. XL contributed reagents, materials, and analysis tools. XL and SZ wrote the paper.

## Funding

This work was funded by the National Natural and Science Foundation of China (No. 31672400).

## Conflict of Interest

The authors declare that the research was conducted in the absence of any commercial or financial relationships that could be construed as a potential conflict of interest.

## Footnotes

Accessed: Sep 26, 2019.

## References

[B1] Alidoust M. Hamzehzadeh L. Rivandi M. Pasdar A. (2018). Polymorphisms in non-coding RNAs and risk of colorectal cancer: a systematic review and meta-analysis. Crit. Rev. Oncol. Hematol. 132, 100–110. 10.1016/j.critrevonc.2018.09.003 30447914

[B2] Aljanabi S. M. Martinez I. (1997). Universal and rapid salt-extraction of high quality genomic DNA for PCR-based techniques. Nucleic Acids Res. 25, 4692–4693. 10.1093/nar/25.22.4692 9358185 PMC147078

[B3] Bharathy N. Ling B. M. Taneja R. (2013). Epigenetic regulation of skeletal muscle development and differentiation. Subcell Biochem. 61, 139–150. 10.1007/978-94-007-4525-4_7 23150250

[B4] Billerey C. Boussaha M. Esquerré D. Rebours E. Djari A. Meersseman C. (2014). Identification of large intergenic non-coding RNAs in bovine muscle using next-generation transcriptomic sequencing. BMC Genomics 15, 499. 10.1186/1471-2164-15-499 24948191 PMC4073507

[B5] Chen H. J. Ihara T. Yoshioka H. Itoyama E. Kitamura S. Nagase H. (2018). Expression levels of brown/beige adipocyte-related genes in fat depots of vitamin A-restricted fattening cattle. J. Anim. Sci. 10.1093/jas/sky240 PMC6127792 29912360

[B6] Chen M. Wang J. Liu N. Cui W. Dong W. Xing B. (2019). Pig SOX9: expression profiles of Sertoli cell (SCs) and a functional 18 bp indel affecting testis weight. Theriogenology 138, 94–101. 10.1016/j.theriogenology.2019.07.008 31319268

[B7] Cui Y. Yan H. Wang K. Xu H. Zhang X. Zhu H. (2018). Insertion/Deletion within the KDM6A gene is significantly associated with litter size in goat. Front. Genet. 9, 91. 10.3389/fgene.2018.00091 29616081 PMC5869274

[B8] Dimicoli-Salazar S. Bulle F. Yacia A. Massé J. M. Fichelson S. Vigon I. (2011). Efficient in vitro myogenic reprogramming of human primary mesenchymal stem cells and endothelial cells by Myf5. Biol. Cell 103, 531–542. 10.1042/BC20100112 21810080

[B9] Fernandes J. C. R. Acuña S. M. Aoki J. I. Floeter-Winter L. M. Muxel S. M. (2019). Long non-coding RNAs in the regulation of gene eExpression: physiology and disease. Noncoding RNA 5, pii: E17. 10.3390/ncrna5010017 30781588 PMC6468922

[B10] Hayashi S. Manabe I. Suzuki Y. Relaix F. Oishi Y. (2016). Klf5 regulates muscle differentiation by directly targeting muscle-specific genes in cooperation with MyoD in mice. Elife. 5, e17462. 10.7554/eLife.17462 27743478 PMC5074804

[B11] Jin C. F. Li Y. Ding X. B. Li X. Zhang L. L. Liu X. F. (2017). lnc133b, a novel, long non-coding RNA, regulates bovine skeletal muscle satellite cell proliferation and differentiation by mediating miR-133b. Gene 630, 35–43. 10.1016/j.gene.2017.07.066 28757453

[B12] Jin Y. Yang Q. Zhang M. Zhang S. Cai H. Dang R. (2018). Identification of a novel polymorphism in bovine lncRNA ADNCR gene and its association with growth traits. Anim. Biotechnol. 30, 159–165. 10.1080/10495398.2018.1456446 29631473

[B13] Kang Z. Jiang E. Wang K. Pan C. Chen H. Yan H. (2019a). Goat membrane associated ring-CH-type finger 1 (MARCH1) mRNA expression and association with litter size. Theriogenology 128, 8–16. 10.1016/j.theriogenology.2019.01.014 30711644

[B14] Kang Z. Zhang S. He L. Zhu H. Wang Z. Yan H. (2019b). A 14-bp functional deletion within the CMTM2 gene is significantly associated with litter size in goat. Theriogenology 139, 49–57. 10.1016/j.theriogenology.2019.07.026 31362196

[B15] Kong L. Zhang Y. Ye Z. Q. Liu X. Q. Zhao S. Q. Wei L. (2007). CPC: assess the protein-coding potential of transcripts using sequence features and support vector machine. Nucleic Acids Res. 35 (Web Server issue), W345–W349. 10.1093/nar/gkm391 17631615 PMC1933232

[B16] Li M. Sun X. Cai H. Sun Y. Plath M. Li C. (2016). Long non-coding RNA ADNCR suppresses adipogenic differentiation by targeting miR-204. Biochim. Biophys. Acta 1859, 871–882. 10.1016/j.bbagrm.2016.05.003 27156885

[B17] Li Y. Chen X. Sun H. Wang H. (2018). Long non-coding RNAs in the regulation of skeletal myogenesis and muscle diseases. Cancer Lett. 417, 58–64. 10.1016/j.canlet.2017.12.015 29253523

[B18] Liu X. F. Ding X. B. Li X. Jin C. F. Yue Y. W. Li G. P. (2017). An atlas and analysis of bovine skeletal muscle long noncoding RNAs. Anim. Genet. 48, 278–286. 10.1111/age.12539 28262958

[B19] Livak K. J. Schmittgen T. D. (2001). Analysis of relative gene expression data using real-time quantitative PCR and the 2(-Delta Delta C(T)) Method. Methods 25, 402–408. 10.1006/meth.2001.1262 11846609

[B20] Lu V. M. Goyal A. Lee A. Jentoft M. Quinones-Hinojosa A. Chaichana K. L. (2019). The prognostic significance of TERT promoter mutations in meningioma: a systematic review and meta-analysis. J. Neurooncol. 142, 1–10. 10.1007/s11060-018-03067-x 30506498

[B21] Nei M. (1973). Analysis of gene diversity in subdivided populations. Proc. Natl. Acad. Sci U S A 70, 3321–3323. 10.1073/pnas.70.12.3321 4519626 PMC427228

[B22] Park S. Y. Yun Y. Kim M. J. Kim I. S. (2014). Myogenin is a positive regulator of MEGF10 expression in skeletal muscle. Biochem. Biophys. Res. Commun. 450, 1631–1637. 10.1016/j.bbrc.2014.07.061 25044114

[B23] Scotto-Lavino E. Du G. Frohman M. A. (2006). 3' end cDNA amplification using classic RACE. Nat. Protoc. 1, 2742–2745. 10.1038/nprot.2006.481 17406530

[B24] Sun X. Li M. Sun Y. Cai H. Lan X. Huang Y. (2016). The developmental transcriptome sequencing of bovine skeletal muscle reveals a long noncoding RNA, lncMD, promotes muscle differentiation by sponging miR-125b. Biochim. Biophys. Acta 1863, 2835–2845. 10.1016/j.bbamcr.2016.08.014 27589905

[B25] Wang X. Yang Q. Wang K. Zhang S. Pan C. Chen H.. (2017). A novel 12-bp indel polymorphism within the GDF9 gene is significantly associated with litter size and growth traits in goats. Anim. Genet. 48, 735–736. 10.1111/age.12617 29023802

[B26] Wang X. Yang Q. Wang K. Yan H. Pan C. Chen H. (2019). Two strongly linked single nucleotide polymorphisms (Q320P and V397I) in GDF9 gene are associated with litter size in cashmere goats. Theriogenology 125, 115–121. 10.1016/j.theriogenology.2018.10.013 30414564

[B27] Xie B. Tassi E. Swift M. R. McDonnell K. Bowden E. T. Wang S. (2006). Identification of the fibroblast growth factor (FGF)-interacting domain in a secreted FGF-binding protein by phage display. J. Biol. Chem. 281, 1137–1144. 10.1074/jbc.M510754200 16257968

[B28] Xu Y. Shi T. Zhou Y. Liu M. Klaus S. Lan X. (2018). A novel PAX7 10-bp indel variant modulates promoter activity, gene expression and contributes to different phenotypes of Chinese cattle. Sci. Rep. 8, 1724. 10.1038/s41598-018-20177-8 29379079 PMC5789009

[B29] Yan P. Luo S. Lu J. Y. Shen X. (2017). Cis- and trans-acting lncRNAs in pluripotency and reprogramming. Curr. Opin. Genet. Dev. 46, 170–178. 10.1016/j.gde.2017.07.009 28843809

[B30] Yang Q. Yan H. Li J. Xu H. Wang K. Zhu H. (2017). A novel 14-bp duplicated deletion within goat GHR gene is significantly associated with growth traits and litter size. Anim. Genet. 48, 499–500. 10.1111/age.12551 28295460

[B31] Yang Q. Zhang S. Li J. Wang X. Peng K. Lan X. (2019). Development of a touch-down multiplex PCR method for simultaneously rapidly detecting three novel insertion/deletions (indels) within one gene: an example for goat GHR gene. Anim. Biotechnol. 10.1080/10495398.2018.1517770 30380974

[B32] Yu X. Zhang Y. Li T. Ma Z. Jia H. Chen Q. (2017). Long non-coding RNA Linc-RAM enhances myogenic differentiation by interacting with MyoD. Nat. Commun. 8, 14016. 10.1038/ncomms14016 28091529 PMC5241866

[B33] Yu X. Wang Z. Sun H. Yang Y. Li K. Tang Z. (2018). Long non-coding MEG3 is a marker for skeletal muscle development and meat production traits in pigs. Anim. Genet. 49, 571–578. 10.1111/age.12712 30294799

[B34] Yue Y. Jin C. Chen M. Zhang L. Liu X. Ma W. (2017). A lncRNA promotes myoblast proliferation by up-regulating GH1. Cell. Dev. Biol. Anim. 53, 699–705. 10.1007/s11626-017-0180-z 28726188

[B35] Zammit P. S. (2017). Function of the myogenic regulatory factors Myf5, MyoD, Myogenin and MRF4 in skeletal muscle, satellite cells and regenerative myogenesis. Semin Cell Dev. Biol. 72, 19–32. 10.1016/j.semcdb.2017.11.011 29127046

[B36] Zhang S. Dang Y. Zhang Q. Qin Q. Lei C. Chen H. (2015). Tetra-primer amplification refractory mutation system PCR (T-ARMS-PCR) rapidly identified a critical missense mutation (P236T) of bovine ACADVL gene affecting growth traits. Gene 559, 184–188. 10.1016/j.gene.2015.01.043 25620159

[B37] Zhao H. Nettleton D. Dekkers J. C. (2007). Evaluation of linkage disequilibrium measures between multi-allelic markers as predictors of linkage disequilibrium between single nucleotide polymorphisms. Genet Res. 89, 1–6. 10.1017/S0016672307008634 17517154

[B38] Zhu M. Liu J. Xiao J. Yang L. Cai M. Shen H. (2017). Lnc-mg is a long non-coding RNA that promotes myogenesis. Nat. Commun. 8, 14718. 10.1038/ncomms14718 28281528 PMC5353601

