# Corrigendum: Novel lncRNA lncFAM200B: Molecular Characteristics and Effects of Genetic Variants on Promoter Activity and Cattle Body Measurement Traits

**DOI:** 10.3389/fgene.2021.770932

**Published:** 2021-10-22

**Authors:** Sihuan Zhang, Zihong Kang, Xiaomei Sun, Xiukai Cao, Chuanying Pan, Ruihua Dang, Chuzhao Lei, Hong Chen, Xianyong Lan

**Affiliations:** ^1^ College of Animal Science and Technology, Northwest A&F University, Yangling, China; ^2^ College of Animal Science and Technology, Yangzhou University, Yangzhou, China

**Keywords:** bovine, lncFAM200B, muscle development, promoter, body measurement traits

In the original article, there was an error. The analyses accession number in the DATA AVAILABILITY STATEMENT section was wrong.

A correction has been made to *DATA AVAILABILITY STATEMENT*:

The detailed information of SNP2 can be found in the European Variation Archive database. Project: PRJEB33081; Analysis Accession: ERZ3224744.

The authors apologize for this error and state that this does not change the scientific conclusions of the article in any way. The original article has been updated.

## Data Availability

The detailed information of SNP2 can be found in the European Variation Archive database. Project: PRJEB33081; Analysis Accession: ERZ3224744.

